# Eating the unknown: Xenophagy and ER-phagy are cytoprotective defenses against pathogens

**DOI:** 10.1016/j.yexcr.2020.112276

**Published:** 2020-11-01

**Authors:** Alessio Reggio, Viviana Buonomo, Paolo Grumati

**Affiliations:** Telethon Institute of Genetics and Medicine (TIGEM), Pozzuoli (NA), Italy

**Keywords:** Autophagy, ER-phagy, Xenophagy, Virophagy, Virus

## Abstract

Autophagy is an evolutionary conserved catabolic process devoted to the removal of unnecessary and harmful cellular components. In its general form, autophagy governs cellular lifecycle through the formation of double membrane vesicles, termed autophagosomes, that enwrap and deliver unwanted intracellular components to lysosomes. In addition to this omniscient role, forms of selective autophagy, relying on specialized receptors for cargo recognition, exert fine-tuned control over cellular homeostasis. In this regard, xenophagy plays a pivotal role in restricting the replication of intracellular pathogens, thus acting as an ancient innate defense system against infections. Recently, selective autophagy of the endoplasmic reticulum (ER), more simply ER-phagy, has been uncovered as a critical mechanism governing ER network shape and function. Six ER-resident proteins have been characterized as ER-phagy receptors and their orchestrated function enables ER homeostasis and turnover overtime. Unfortunately, ER is also the preferred site for viral replication and several viruses hijack ER machinery for their needs. Thus, it is not surprising that some ER-phagy receptors can act to counteract viral replication and minimize the spread of infection throughout the organism. On the other hand, evolutionary pressure has armed pathogens with strategies to evade and subvert xenophagy and ER-phagy. Although ER-phagy biology is still in its infancy, the present review aims to summarize recent ER-phagy literature, with a special focus on its role in counteracting viral infections. Moreover, we aim to offer some hints for future targeted approaches to counteract host-pathogen interactions by modulating xenophagy and ER-phagy pathways.

## 1 A sneak-peek into the complexity of macroautophagy

The term autophagy refers to a non-selective lysosome-dependent degradation process that cells normally exploit for intracellular housekeeping and macromolecule recycling. Specifically, autophagy ensures a baseline removal of damaged or proteotoxic intracellular structures (i.e. organelles and protein aggregates, respectively) as well as the availability of a ready-to-use pool of amino acids, lipids and other precursors that cells can direct towards biosynthetic pathways. Autophagic flux is cell specific and is mainly dictated by the needs of each cell and tissue [1]. Indeed, cells from post-natal tissues can modulate their basal autophagic flux to properly respond to different stimuli [2,3] including unbalance of nutrient availability [4], growth factor fluctuations [5,6] and pathogen infections [7]. This degradative process relies on the capability of double-membraned vesicles, known as autophagosomes, to encapsulate cytoplasmic materials prior to their delivery to lysosomes [8]. In higher eukaryotes, mature autophagosomes are formed by the closure of cup-shaped phagophores, mainly originating at specialized ER sites, called omegasomes [9]. Here, phagophore expansion occurs and cytosolic portions are progressively trapped as cargo. Finally, the double-membraned autophagosome fuses with a lysosome where the engulfed materials are processed by hydrolytic enzymes [10].

From a molecular point of view, autophagy induction requires the participation of multiple protein partners and complexes that in concert cooperate to facilitate phagophore nucleation, elongation and closure. Inhibition of the mechanistic target of rapamycin (mTOR) is considered to be the main intracellular signal responsible for autophagy induction [4,11]. Upon mTOR blockade, the Unc-51-like kinase 1/2 (ULK1/2) complex, consisting of ULK1/2, autophagy-related protein 13 (ATG13), FAK family kinase interacting protein of 200 kDa (FIP200) and ATG101 [12,13], is activated and is recruited to the omegasome [14]. Here, ULK1/2 complex triggers phagophore nucleation by recruiting components of the class III phosphatidylinositol-3-kinase (PI3KC3) complex I [15]. Once activated, PI3KC3 complex I components, consisting of class III PI3K, Beclin 1, ATG14, vacuolar protein sorting 34 (VPS34) and activating molecule in Beclin 1-regulated autophagy protein 1 (AMBRA1), cooperate to increase local concentrations of phosphatidylinositol-3-phosphate (PI3P), which in turn acts to recruit PI3P-binding autophagy effector proteins to the newly formed phagophore membrane [[15], [16], [17]]. Among them is WIPI2, that acts as a docking site pivotal for the formation of the ATG5–12–16 complex on the outer phagophore membrane [[18], [19], [20]]. The ATG5–12–16 complex then promotes ATG3-mediated conjugation of activated ubiquitin-like ATG8 family members [21]. Phosphatidylethanolamine (PE)-anchored (i.e. lipidated) forms of microtubule-associated protein light chain 3 (LC3) proteins and γ-aminobutyric acid receptor-associated proteins (GABARAPs) are present on the inner and outer membranes of newly formed phagophores, thus contributing to the elongation and then sealing of the phagophore [22,23]. At this stage, Golgi complexes, plasma membrane, recycling endosomes, mitochondria and ATG9-enriched vesicles provide additional sources of membrane for growing autophagosomes [3]. The final stages of autophagy, autophagosome maturation and cargo destruction, require lysosomal delivery and fusion [24] which are accomplished with the help of specialized machinery.

## 2 Selective forms of autophagy

Starvation-induced autophagy processes large amounts of cytosolic materials that are subsequently disassembled in-bulk. This cellular task appears to lack selectively, and the encapsulated materials are randomly trapped during phagophore elongation and closure. However, our understanding of autophagy has significantly expanded over the past several years, uncovering highly regulated and somewhat interconnected signaling pathways governing targeted forms of autophagy [25]. Selective autophagy relies on the existence of specialized autophagy receptors that drag labelled cargos into autophagosomes [25,26]. Receptors directly bind to LC3/GABARAP on autophagosomal membranes and are themselves degraded along with their cargo within lysosomes. Specifically, autophagy receptors identify an “eat-me” signal, mostly composed of ubiquitin chains, on cargo (e.i, p62, OPTN) [[27], [28], [29]] [[27], [28], [29]] [[27], [28], [29]] or are themselves resident proteins of the targeted organelle (e.i, NIX, FAM134B) [30,31]. Cargo receptors also possess an LC3-interacting region (LIR, with a consensus [W/F/Y]‐X1‐X2‐[I/L/V]) or GABARAP interaction motif (GIM, [W/F]‐[V/I]‐X2‐V), which is often located in the long unstructured sequences of these receptors, and are recognized by LC3s and GABARAPs respectively [26]. As a result, autophagy receptors and cargos are simultaneously engulfed by nascent autophagosome and then degraded in the hydrolytic milieu of the lysosome [32]. Therefore, by coupling a rich repertoire of autophagy receptors with a highly conserved core autophagic machinery, cells exploit this process for the selective removal of defined cellular or subcellular components including misfolded protein aggregates (aggrephagy), damaged mitochondria (mitophagy), peroxisomes (pexophagy), ribosomes (ribophagy) and endoplasmic reticulum (ER-phagy) [26]. The existence of a precise and selective autophagic process depends on several regulatory mechanisms which are devoted to control the expression, localization and activity of autophagy receptors. Specifically, to maintain an appropriate basal level of autophagy receptors without triggering their accumulation over time, most autophagy receptors and adaptors undergo constant turnover by autophagy even in their unloaded state [33]. Moreover, post-translational modifications (PTMs) are involved in these regulatory circuits too. Consistently, phosphorylation is a highly conserved and rapidly deployable PTM that cells can use to control autophagy. For example, phosphorylation of the LIR domain of BNIP3 promotes its binding with LC3 modifiers [34]. Similarly, phosphorylation of p62 and OPTN increases their affinity for ubiquitin chains and LC3 during salmonella infection [28]. Now, great research efforts are concentrated on the identification of PTMs that govern autophagy selectivity and regulation in different contexts [35].

In addition to its role as a scavenger, autophagy is also considered an ancient defense system that cells use to counteract bacterial and viral infections. Xenophagy is a specialized form of autophagy dedicated to “eating” foreign organisms that are potentially harmful for cells [36,37]. Some autophagy receptors, like p62/SQSTM1 and OPTN, can mediate the selective recognition and removal of intracellular bacteria [28,29]. In the same way, ER-phagy, atop of its intrinsic role in controlling ER shape and homeostasis, acts to limit the spread of viruses [38,39]. This is a particularly valuable cellular defense strategy since viruses tend to use the ER as their primary site for replication and accumulation. In the next sections, we will focus our discussion on xenophagy and ER-phagy as first lines of the innate immune response, emphasizing the molecular strategies that pathogens have evolved to escape these cytoprotective controls.

## 3 Xenophagy: eating the unknown

**Fig. 1:**
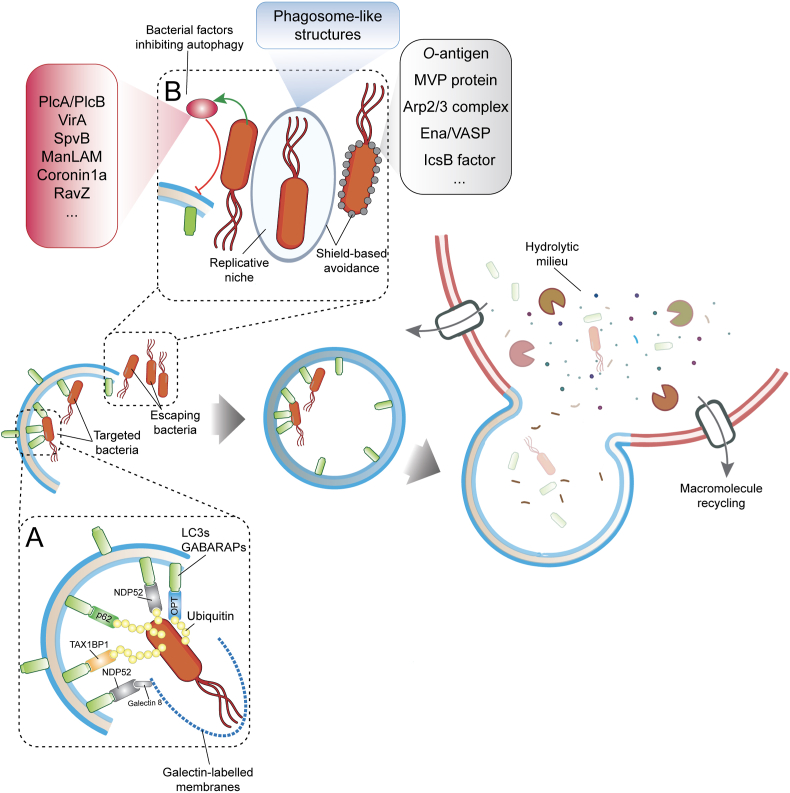
**Xenophagy restricts intracellular pathogen infections**. (**A**) Autophagy adaptors are devoted to the recognition of ubiquitin chains and galectins that mark invading bacteria and damaged bacterial phagosome-like membranes, respectively. (**B**) Intracellular pathogens can escape the autophagy-dependent destruction either eluding the autophagy machinery or inhibiting autophagosome maturation.

Xenophagy targets intracellular bacteria for lysosomal degradation, and acts as an important innate immune defense that mammalian cells can adopt to cope with potential life-threatening pathogens. Xenophagy is controlled in time and space and its activation requires surveillance mechanisms that are able to recognize invading organisms. Intracellular membrane rupture is one of the first events that expose bacteria to the host cytosol. Once exposed, pathogens are promptly labelled with ubiquitin chains, while the disrupted membranes are recognized by Galectin-8. Such post-translational modifications act as docking sites for specialized autophagy receptors: p62/SQSTM1, OPTN, NDP52 and TAX1BP1 [28,[40], [41], [42]](Fig. 1A). These autophagy receptors interact with LC3/GABARAP to link the decorated membrane and the recognized bacteria to the autophagy machinery and deliver them to lysosomes for clearance (Fig. 1A). Consistently, deletion of autophagy genes (i.e. ULK1, Beclin 1, ATG5, ATG7) causes the loss of xenophagy-dependent control of bacterial replication in cultures cells and animal models [[43], [44], [45], [46]]. Murine strains, where Atg5 is conditionally deleted in myeloid-derived cells (Atg5^fl/fl^ LysM-Cre), succumb by *Mycobacterium tuberculosis* infection within a few days of infection [45,47]. Similarly, *C. elegans* with defective autophagy genes exhibit vulnerability to bacterial infections [48].

While xenophagy provides the first line of innate defense, many bacteria evolved anti-autophagy strategies to efficiently replicate inside cells and spread across tissues undetected and unscathed. Such strategies can involve avoidance of cellular surveillance mechanisms, impairment of phagophore formation as well as subversion of autophagy machinery (Fig. 1B).

A common strategy used by several pathogens to escape xenophagy is to shield themselves from being targeted by ubiquitin chains or being recognized by autophagy proteins. *Francisella tularensis* is a clear-cut example of this shield-base escape. The surface of *F. tularensis* is coated with polysaccharidic O-antigens that protect it against poly-ubiquitination and xenophagy-dependent destruction [49]. Furthermore, the antioxidant super-oxide dismutase of *F. tularensis* can inhibit ROS-dependent induction of xenophagy, while impairing LC3-associated phagocytosis (LAP) during infection of cultured murine macrophages [50]. In a similar manner, *Listeria monocytogenes* can avoid xenophagy by decorating its surface with the host's Major vault protein (MVP) through the activity of the factor InlK [51]. Moreover, by exploiting the virulence factor ActA, *L. monocytogenes* can recruit the Arp2/3 complex and Ena/VASP to the bacterial surface, thus preventing its ubiquitination and autophagy [52,53] (Fig. 1B). Although slower than their preferred cytosolic replication, *L. monocytogenes* can proliferate in spacious *Listeria*-containing phagosomes (SLAPs) that are decorated by LC3 and LAMP1 [54]. Using the same strategy, *Shigella flexneri* is able to escape xenophagy by secreting bacterial IcsB effector, which disguises a region of the IcsA protein on the bacterial surface [[55], [56], [57], [58]]. In this way, IcsA cannot be recognized by ATG5, thereby blocking *S. flexneri* recruitment to the phagophore [55].

While the above reported examples demonstrate the advantages of stealth-mode in escaping intracellular surveillance mechanisms, a successful bacterial replication requires additional strategies that interfere with xenophagy pathways at multiple levels. In this context, coupled to their shield-based strategy, *L. monocytogenes* dampens autophagosome formation and maturation by lowering local concentrations of PIP3 through the induction of bacterial PlcA and PlcB phospholipases [59,60] (Fig. 1B). Similarly, *S. flexneri*, can prevent early steps of phagophore nucleation and elongation through the bacterial protein VirA. VirA is a GTPase-activating protein (GAP) that inhibits Rab1, whose activity is crucial for phagophore nucleation and for the recruitment of LC3 to the surface of *S. flexneri* [58]. Similar to *L. monocytogenes* and *S. flexneri*, *Salmonella thyphimurium* can also suppress autophagosome formation by depolymerizing actin through the activity of the bacterial protein SpvB [61].

Another strategy implemented by pathogens is to interfere with autophagosome maturation while subverting the autophagic machinery to help increase their chances for survival and replication. In the case of *Mycobacterium tuberculosis* infection, several avoidance strategies are set in motion in order to ensure successful colonization of the host organism. *M. tuberculosis* efficiently prevents autophagosome fusion by lowering PI3P levels through the activity of mannose-capped lipoarabinomannan (ManLAM) [62] (Fig. 1B). Moreover, in a ManLAM-dependent mechanism, *M. tuberculosis* inhibits Ca^2+^ influx thereby impairing upstream mTOR and ULK1 signaling that is required in the early steps of autophagy [62]. Notably, *M. tuberculosis* also exploits the bacterial factor Coronin1a to impair autophagosome formation [63]. Using similar subversion strategies, *Legionella pneumophila* blocks autophagosome acidification by irreversibly deconjugating LC3s/GABARAPs via the effector protein RavZ [64]. In parallel, by translocating the effector protein sphingosine-1 phosphate lyase (LpSpl), *L. pneumophila* can also limit host sphingosine biosynthesis thereby curtailing autophagy/xenophagy [65]. To obtain a continuous flux of nutrients, *Anaplasma phagocytophilum* enhances autophagosome formation, via the effector Anaplasma translocated substrate 1 (Ats-1), while replicating inside nutrient rich double-lipid bilayer membranes associated with LC3, Beclin 1 and Atg6 but devoid of lysosomal markers [66]. Likewise, *Coxiella burnetii* survives and replicates in acidified vesicles that colocalize with LC3 during infection of epithelial cells and macrophages [67,68]. Similarly, *Yersinia pseudotuberculosis*, *Y. pestis*, *Staphylococcus aureus*, *Serratia marcescens* and *Brucella abortus* replicate intracellularly in autophagosomes or autophagosome-like structures and prevent their maturation and fusion with lysosomes [[69], [70], [71], [72], [73]]. Of note, cell colonization by invading bacteria is likely the result of combined “espionage techniques” against xenophagy.

## 4 Targeting intracellular pathogens by modulating the autophagy/xenophagy flux

Modulation of the autophagy flux is emerging as a feasible strategy to counteract microbial infections and some pharmacological agents with these properties have entered clinical trials. Inhibiting autophagy with 3-methyladenine arrests the growth of *Anaplasma phagocytophilum* [74]. Using an opposite modulation, the PDK1 inhibitor AR-12 induces autophagy and efficiently eliminates *Salmonella typhimurium* in murine macrophages and *Francisella tularensis* in human leukemic THP-1 macrophages [75,76]. The EGFR inhibitor Gefitinib, used as anti-cancer agent, is also effective in limiting the spread of *M. tuberculosis* infection by forcing autophagy activation [77]. However, the evident disadvantage of these pharmacological approaches is their promiscuity towards other molecular targets, therefore more-specific agents that more directly modulate autophagy are continuously explored. Of note, cell-permeable autophagy-inducing peptides are promising candidates. Among them Tat–Beclin 1, which is known to stimulate autophagy by interfering with the negative autophagy regulator GAPR-1^78^. Tat–Beclin 1 administration boosts autophagy whilst limiting the replication of the Δ*ActA* strain of *L. monocytogenes* in infected cultured macrophages [78].

While these approaches are promising for counteracting intracellular bacterial replication by modulating autophagy in opportune *in vitro* scenarios and murine strains, future research should clarify potential translational applications of these treatments. Future efforts will be also focused on accelerating research platforms involved in the discovery of more-effective targets in host-directed therapies that can be coupled with newly engineered perturbagens that limit side-target effects in system-wide autophagy manipulation approaches.

## 5 Autophagy as a first line of defense against viral infections

Viruses are generally considered to be non-living organisms capable of infecting hosts from every branch of the tree of life. Approximately 200 species of viruses are known to infect and cause disease in humans [79]. To counteract potentially lethal viral illness, cells have evolved virus‐specific controls and innate immune responses aimed at counteracting and eliminating viruses [80]. In the past decade autophagy has emerged as a pivotal cellular defense mechanism that limits viral infection and this selective form of autophagy is called virophagy [81]. Some autophagy-dependent strategies against viruses have been characterized in-depth in *Drosophila melanogaster*, since autophagy is one of the major components governing flies’ immunity [82]. Here, the autophagic machinery processes and delivers antigens to Toll-Like Receptors (TLRs) upon vesicular stomatitis virus (VSV) infection, thus stimulating an immune response [83]. Using a similar mechanism Toll-7 and MyD88 signaling have been demonstrated to hamper the replication of the Rift Valley fever virus (RVFV) in flies and mammals, respectively [84]. In mammals, one of the best characterized antiviral roles, directed by the autophagy pathway, is the processing and subsequent delivery of intracellular viral antigens to the peptide grooves of the major histocompatibility complex (MHC) class I upon infection by herpes simplex virus type 1 (HSV-1) [85]. Autophagy also delivers cytosolic proteins for MHC class II presentation which enhances T cell activation [86]. However, similarly to bacteria, viruses have evolved subversive mechanisms against autophagy, increasing their chances for replication and viral pathogenesis. In fact, influenza virus [87], coronaviruses [88], coxsackievirus [89], poliovirus [90], hepatitis C virus (HCV) [91,92] and DENV [93] are known to largely affect autophagic machinery. Influenza A virus (IAV) inhibits autophagosome-to-lysosome fusion [87]. As a consequence, autophagosomes containing viruses accumulate in infected cells allowing viral replication and virion assembly [87]. Newcastle disease virus (NDV) can trigger autophagy in U251 glioma cells to enhance viral replication [94]. Of note, pharmacological or genetic manipulation of the autophagy pathway efficiently halts NDV replication [95]. In contrast, HIV exerts a double-fronted assault on autophagy. During the initial stages of infection, HIV induces autophagosome formation while blocking their maturation and fusion during the late stages of viral replication [96,97]. This results in a massive accumulation of immature autophagosomes, providing membranes to be used as scaffolds for HIV virion assembly [98]. A similar strategy is used by the measles virus (MeV), where increasing autophagic flux results in increased viral replication inside cells [99,100]. Interestingly, MeV utilizes autophagy receptors NDP52 and TAX1BP1, possibly through direct binding by viral effector proteins, for the maturation of a subset of MeV-containing autophagosomes that are required for viral replication. With this in mind, our understanding of the autophagic mechanisms restricting viral infections is far from complete and new intrinsic anti-viral mechanisms continue to be uncovered. Recently, a selective form of autophagy that specifically targets the ER (ER-phagy), has been implicated in controlling cellular fitness and the ability of cells to adapt to different stressful stimuli. Notably, the ER is also one of the favorite sites for viral replication, accumulation, and virion assembly [101]. Thus, ER-phagy can represent a particularly valuable form of anti-virus response through the combination of a highly specialized receptor repertoire coupled with generic autophagy machinery. In the next section, we will discuss ER-phagy and its anti-viral role more in-depth.

## 6 ER-phagy as a specialized innate immune response

**Fig. 2:**
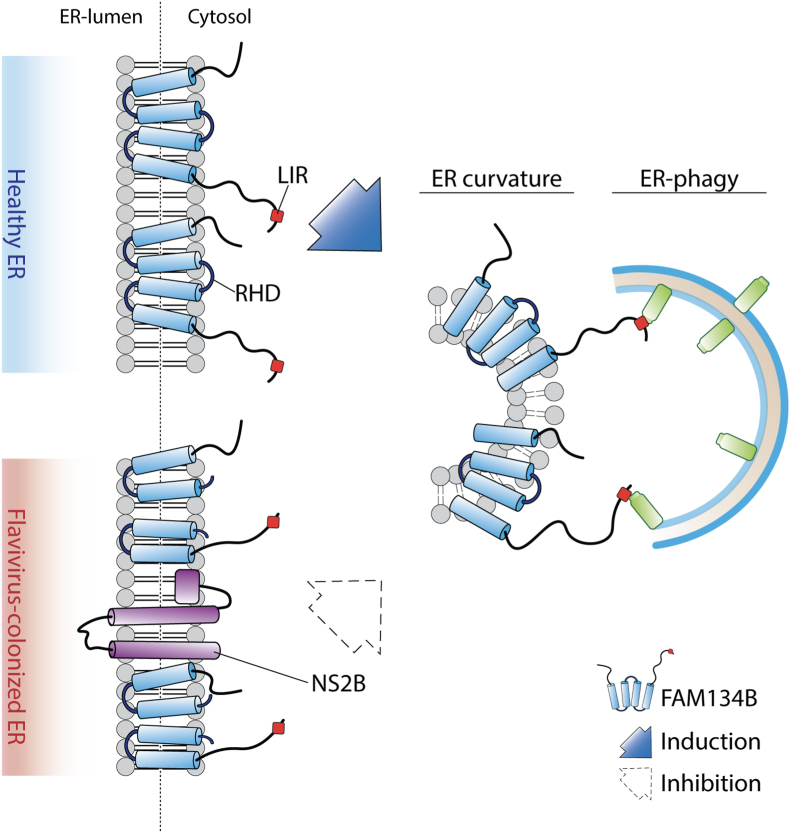
**Flavivirus efficiently replicate in ER subdomains by subverting FAM134B-dependent ER-phagy**. NS2B protein efficiently cleaves the reticulum homology domain of FAM134B, thus impairing the ER-phagy dependent clearance of virally-colonized ER subdomains.

The ER is a membranous organelle, which develops a dynamic and elaborate network, originating from the nuclear membrane, and spanning throughout the cytosol [102]. Its morphology is complex, subject to constant remodeling and, dependent on function, assumes distinct shapes. While ER tubules are very dynamic and constantly elongate, retract, fuse and slide along the cytoskeleton [[102], [103], [104]], ER sheets are less mobile but enlarge in response to ER stress (like calcium imbalance, accumulation of protein aggregates, viral infections) and shrink back to their original size after the stress is resolved [105]. Cells that fail to properly support ER remodeling and turnover are unable to respond to cellular needs or resolve ER stress. This is a common reason for the development of human diseases [106,107]. ER remodeling as well as its constant turnover is mediated by a cellular recycling pathway known as ER-phagy. The mammalian proteins FAM134B, SEC62, RTN3, CCPG1, ATL3 and TEX264 have been identified as autophagy receptors that mediate the coupling of ER fragments to autophagic membranes, thereby participating in the basal turnover of ER, re-shaping after ER expansion upon stress, as well as lysosomal degradation of ER protein aggregates [31,[108], [109], [110], [111], [112], [113]]. These ER-phagy receptors are specialized for their respective ER subdomains and/or specific stress responses. FAM134B is an intra-membrane ER-resident protein which plays a key role in the basal turnover of ER sheets. It harbors a reticulon homology domain (RHD) that can curve ER membranes (Fig. 2) and generates small vesicles. Its LIR domain enables it to bind to mATG8s which promotes the delivery of ER membranes to lysosomes. Absence of FAM134B promotes ER expansion and results in ER stress, affecting neuron survival, and leading to neuropathies [31,114]. During infection, ER associated degradation is exploited by viruses for replication and immune response evasion. Viruses assemble and mature in ER compartments during their infection cycles; therefore, the elimination of specific portions of the ER, via the lysosomal system, is an innate antiviral strategy adopted by host cells to counteract viruses and directly eliminate their proliferative site [115]. Absence of FAM134B results in ER expansion [31] which is also a consequence of flavivirus infection, as these viruses use the ER as a proliferative niche [116,117] (Fig. 2). However, how FAM134B specifically recognizes viral replicative sites within the ER to mediate their elimination remains uncharacterized. Nevertheless, Zika (ZIKV), Dengue (DENV) and West Nile viruses appear to antagonize ER-phagy by directly targeting FAM134 B[38,39] (Fig. 2). Their NS3 virally-encoded protease and its cofactor NS2B cleave FAM134B within its RHD [39], thus impairing FAM134B's intrinsic ability to generate ER vesicles [114] (Fig. 2). Ablation of FAM134B supports Dengue and Zika virus infections, providing direct proof for its pivotal role in mitigating viral infections. Therefore, the cleavage of FAM134B directly benefits flavivirus proliferation [38,118]. Indeed, depletion of the autophagy regulator BPIFB3 enhances FAM134B ER-phagy and impairs flavivirus replication [119]. This suggests that BPIFB3 may have an indirect role in ER remodeling and could regulate DENV and ZIKV proliferation. Of note, BPIFB3 also negatively influences enterovirus replication [119]. In addition, Flavivirus also escapes ER-phagy by targeting RTN3, which is another ER-phagy receptor that can directly fragment ER membranes. Consistently, Flavivirus-encoded NS3A protein which binds RTN3 in order to hijack ER membranes, can generate a replication permissive niche [120]. In addition to flavivirus, FAM134B and RTN3 can affect the replicative status of other types of pathogens. In fact, Ebola virus replication is limited by FAM134B-dependent ER-phagy [38], while RTN3 binding to the NS4B protease of hepatitis C impairs its viral proliferation [121]. Of note, especially in the current days, positive-strand RNA viruses, like SARS-CoV, can also reorganize host cell membranes to build their replicative niche and likely hide RNA replication from antiviral defense mechanisms. Coronaviruses generate a large number of isolated “double-membrane vesicles” (DMVs) whose origin remains obscure. They could derive from ER, late endosome, autophagosome or the secretory pathway [122,123]. However, DMVs are not isolated structures and tend to integrate into a unique reticulo-vesicular network of shaped ER membranes. Therefore, ER is a major source for viral membrane networks, in the case of coronavirus as well [124]. A recent proteomic analysis unraveled the mouse hepatitis virus (MHV), a murine coronavirus, microenvironment and highlighted the functional importance of the ER-Golgi trafficking pathway, ubiquitin and autophagy catabolic systems and translation initiation factors [125]. Even if coronaviruses replication is not clearly affected by macro-autophagy, DMVs are labelled with nonlipidated LC3-I [88]. Of note, FAM134B and its transcriptional regulator TFEB [126] were among the host proteins identified to significantly impact MHV replication [125]; therefore, ER-phagy could play a role in the replication of coronavirus. Viruses are not the only infectious pathogens that target ER membranes. Host cells activate ER-phagy as a response mechanism to alleviate ER stress after Gram-positive bacterial infection. Although the molecular mechanisms are not completely elucidated, upon infection c-di-AMP/STING dependent ER stress seems to be apical to mTORC1 inactivation and consequent autophagy induction [127]. Of note, the SidE enzyme family of the Gram-negative bacteria, *Legionella pneumophila,* promote the non-canonical phosphoribosyl-linked (PR) serine ubiquitination of several ER proteins in order to remodel the ER membrane network and create a proliferative vacuole. Intriguingly, among the PR-ubiquitinated proteins, the ER-phagy receptors RTN3 and Tex264 as well as FAM134C have been recently identified, implicating their direct involvement in ER remodeling in response to pathogen infection [109,111,113,128].

## 7 Conclusions

Several types of viruses and bacteria remodel ER membranes and hijack ER machinery in order to generate an auspicious proliferative environment. The molecular mechanisms adopted by pathogens to conquer the ER are not sufficiently understood, creating obstacles for the development of new/alternative therapeutic approaches. In some cases, like the SARS-CoV-2 pandemic, there are no established therapies nor vaccines to counteract the infection. A major hurdle is our lack of understanding of SARS-CoV-2 biology. Coronaviruses remodel the ER of host cells in order to create their proliferation niche; however, their adopted molecular mechanisms still remain elusive. Further studies are needed to elucidate the role of autophagy and ER-phagy in host cell immunity. These studies will improve our knowledge of how cells counteract viral and bacterial infections and will contribute to the development of novel molecules that specifically act to control ER membrane dynamics to tackle pathogen infections.

## CRediT authorship contribution statement

**Alessio Reggio:** Writing - review & editing. **Viviana Buonomo:** Writing - review & editing. **Paolo Grumati:** Conceptualization, Writing - review & editing.

## Declaration of competing interest

There are no conflict of interest.
